# Immune challenges and pathogen risks in edible insects: safeguarding health in space life-support systems

**DOI:** 10.3389/fphys.2025.1628696

**Published:** 2025-09-03

**Authors:** Annette Bruun Jensen, David Copplestone, Roberto Guidetti, Martina Heer, Paola Pittia, Åsa Berggren

**Affiliations:** ^1^ Department of Plant and Environmental Sciences, University of Copenhagen, Frederiksberg, Denmark; ^2^ Biological and Environmental Sciences, University of Stirling, Stirling, United Kingdom; ^3^ Department of Life Sciences, University of Modena and Reggio Emilia, Modena, Italy; ^4^ IU International University of Applied Sciences, Bad Reichenhall, Germany; ^5^ Institute for Nutritional and Food Sciences, University of Bonn, Bonn, Germany; ^6^ Department of Bioscience and Technology for Food Agriculture and Environment, University of Teramo, Teramo, Italy; ^7^ Department of Ecology, Swedish University of Agricultural Sciences, Uppsala, Sweden

**Keywords:** edible insects, space agriculture, food, microbiology, pathogen virulence

## Abstract

As space agencies progress toward long-duration missions and extraterrestrial colonisation, Bioregenerative Life Support Systems (BLSS) have become central to achieving closed-loop sustainability. Edible insects offer a highly efficient protein source suited for BLSS integration, yet the unique stressors of spaceflight, microgravity, ionising radiation, and limited microbial exposure, pose significant risks to insect immunity and pathogen dynamics. This review synthesises current research on insect immune function, microbiome stability, and disease susceptibility under space-relevant conditions, highlighting vulnerabilities introduced by physical, nutritional and behavioural stressors. We emphasise species-specific immune traits, life stage- and sex-dependent responses, and the contribution of natural behaviours and transgenerational immunity to colony resilience. Further, we examine the synergistic effects of the space environment and high-density rearing on pathogen transmission and virulence evolution. Mitigation strategies, including environmental controls, probiotic interventions and biosensor-based health monitoring, are discussed. By identifying critical knowledge gaps, particularly concerning immune suppression under microgravity and radiation, density-driven pathogen evolution, and the stability of behavioural immunity, we propose system-level responses to support robust insect health. Our synthesis advances the framework for designing resilient, health-optimised insect rearing systems for future space missions and terrestrial applications. Ensuring insect immune competence will be essential for ecological stability and food security in extraterrestrial environments.

## 1 Introduction

As humanity prepares for long-duration space missions and potential colonisation of extraterrestrial environments, developing closed-loop life support systems that can sustain human life independently from Earth is critical (ESA, 2022; NASA, 2023). Bioregenerative Life Support Systems (BLSS) are designed to integrate biological components such as plants, animals and microorganisms to recycle air, water, and waste while producing food and maintaining environmental stability (Berggren et al., 2025). Among candidate organisms for space-based protein production, insects stand out due to their high feed conversion efficiency, minimal space and water requirements, rapid growth, and nutritional richness (Berggren et al., 2019). Among insects, yellow mealworms (*Tenebrio molitor*) and house crickets (*Acheta domesticus*) are currently two of the primary species considered for BLSS, due to amongst other things their well-documented nutritional profiles, appreciated flavour and the established rearing knowledge of the species. The role of insects in BLSS has gained increasing scientific interest due to the targeted efforts in space exploration (ESA, 2022; Berggren et al., 2025). Successfully rearing viable insect populations in space will require both fundamental research and targeted development. For many candidate species, key thresholds and optimal conditions remain unknown. However, terrestrial data provide a starting point. Most edible insect species can be reared in controlled modules maintained at approximately 27 °C–30 °C and 50%–70% relative humidity, using plant-based feed (van Huis et al., 2013; Berggren et al., 2019). Ground-based experiments show that silkworm larvae (*Bombyx mori*) can be reared in controlled and enclosed modules (Tong et al., 2011). Under comparable Earth-based conditions, house crickets can reach harvest size within 8 weeks while requiring less than 2 L of water per kilogram of protein—an important logistical advantage in BLSS. Insects currently approved for consumption within the EU provide 53%–70% digestible protein along with essential micronutrients, making them a valuable complement to plant-based dietary staples (Rumpold and Schlüter, 2013).

**FIGURE 1:**
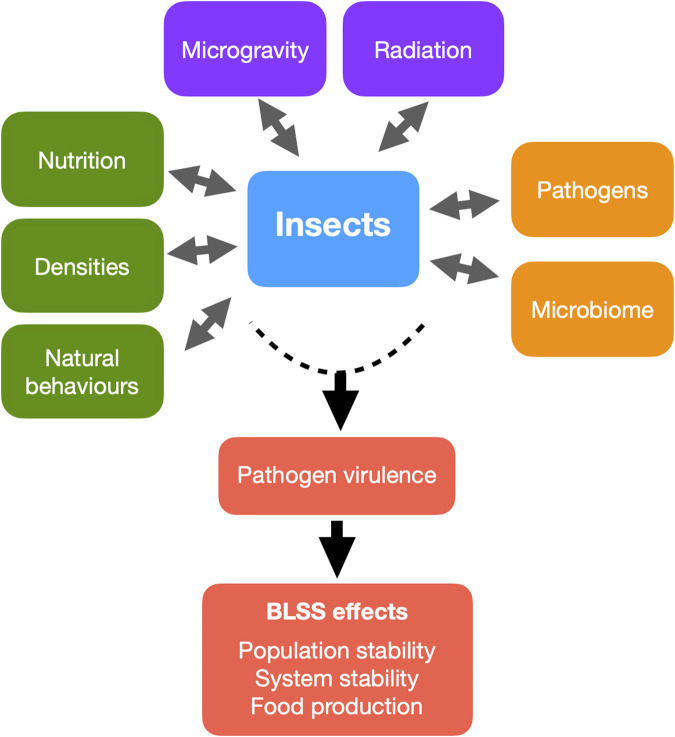
Main factors influencing the health of insects reared in Bioregenerative Life Support Systems in space. These factors can act individually or in interaction with one another to affect immune competence, pathogen risks and system resilience.

However, space introduces novel stressors that challenge the viability of insect rearing. Key environmental variables such as microgravity and ionising radiation can significantly affect insect physiology, especially immune function (Iyer et al., 2022). For instance, space-flown *Drosophila melanogaster* have demonstrated suppressed innate immune responses and reduced expression of antimicrobial peptides, increasing their vulnerability to pathogens (Marcu et al., 2011; Taylor et al., 2014). Compounding this risk, studies show that pathogenic bacteria such as *Serratia marcescens* exhibit increased virulence when cultured in microgravity environments, potentially overwhelming weakened insect immune systems (Gilbert et al., 2020; Gilbert et al., 2022). Furthermore, sterile or microbially reduced space habitats may inadvertently contribute to immune dysregulation by limiting natural microbial exposure, a phenomenon also observed in human astronauts (Mermel, 2013). These findings underscore the importance of understanding host-pathogen-environment interactions under space conditions. The resilience of insects in BLSS, and by extension the success of extraterrestrial missions, depends heavily on our ability to maintain insect health, stable populations and productivity. This review aims to synthesise current knowledge on insect immunity and pathogen interactions in the context of spaceflight, with a particular focus on edible insect species relevant to BLSS. We identify critical research gaps and outline future strategies for monitoring, mitigation, and system design to ensure robust insect health in long-duration missions. This review synthesizes current knowledge on how five major spaceflight-relevant stressors: microgravity, radiation, nutrition, rearing density, and behavioural constraints, interact to affect insect health, with implications for immune competence, pathogen risks, and system resilience in BLSS (Figure 1).

## 2 Insect immunity: a foundation for health in space

The first line of defence in insects is the exoskeleton, serving as a critical physical barrier. Once breached, insects initiate a cascade of cellular and humoral immune responses (Evans et al., 2006). The immune responses are of three different types. First, melanisation through the pro-phenoloxidase cascade generates microbe-toxic melanin (Cerenius and Söderhäll, 2004). Second, haemocytes drive encapsulation, isolating larger pathogens. Third, antimicrobial peptides (AMPs) such as defensins and attacins, lysate invading microbes (Bulet and Stöcklin, 2005). Unlike vertebrates, insects lack adaptive immunity and rely solely on the mechanisms encapsulation, melanisation and antimicrobial peptide production (Vilmos and Kurucz, 1998). Spaceflight has been shown to suppress all three responses in *Drosophila*, highlighting them as key targets for immune support strategies in BLSS environments (Marcu et al., 2011). The ability of an individual of a species to use these defences is a result of both genetic, evolutionary and environmental factors (Berggren, 2009; Berggren and Low, 2009). The immune functions are central to survival in terrestrial environments and become even more vital under the compounded stressors encountered in space-based systems.

### 2.1 The role of the insect microbiome in immune resilience

In terrestrial settings, the gut microbiota plays a key role in maintaining immune equilibrium, aiding in nutrient metabolism, immune priming, and pathogen exclusion (Douglas, 2015). This mutualistic relationship influences larval development, environmental responsiveness, and even behaviour. However, in sterilised, microbially constrained conditions of space habitats, these dynamics may be disrupted, leading to dysbiosis and increased susceptibility to opportunistic infections. Reduced microbial exposure and sanitised feed can compromise immune signalling pathways, a phenomenon observed in astronauts (Mermel, 2013) and likely relevant to insects. Though studies in edible insect species under space conditions remain sparse, findings from other systems suggest that gut microbes can affect how insects spread disease, compete with pathogens, and secrete antiviral agents (Engel and Moran, 2013). Experiments already shown that microgravity disrupts host–microbe relationships: it alters gene expression in symbiotic microbes and increases *Serratia* virulence in *Drosophila* (Casaburi et al., 2017; Gilbert et al., 2020). Future measures may include tailored probiotic supplementation and co-cultivation with beneficial microbial communities to restore microbial balance and reinforce immune function within BLSS. Probiotics have been seen to boost mealworm performance, although the gains are context dependent. In *T. molitor*, an indigenous *Pediococcus pentosaceus* promoted larval growth, whereas a Bacillus strain had the opposite effect (Lecocq et al., 2021), while the endogenous *P. pentosaceus* and an exogenous *Enterococcus faecium* strain enhanced metamorphosis and fungal resistance (Dahal et al., 2022). Before BLSS use, candidate strains should pass genome screens for transferable resistance or toxins (van der Fels-Klerx et al., 2018) and used in micro-g/radiation trials confirming they do not raise *Salmonella* or *Listeria* risk for humans (Gałęcki et al., 2023). Carrying out multigenerational tests would also verify whether any benefits persist without nutritional or reproductive costs.

### 2.2 Species-specific immune responses in edible insects

Species- and stage-specific differences in immune traits are critical to designing resilient BLSS systems. In *A. domesticus*, immune system development shows that phenoloxidase (that is needed to produce melanin) activity increases after metamorphosis, whereas encapsulation responses remain consistent, indicating that immune function varies across life stages (Piñera et al., 2013). Similarly, *T. molitor* displays cuticle colour-dependent immune investment, with darker morphs exhibiting greater phenoloxidase activity and pathogen resistance (Armitage and Siva-Jothy, 2005; Barnes and Siva-Jothy, 2000). These traits are modulated by environmental stressors such as diet, crowding and light. For example, food deprivation suppresses antimicrobial defences (Siva-Jothy and Thompson, 2002). Crowding during the final nymph stage increases phenoloxidase activity but has little effect on survival to adulthood (Piñera, 2012). In Piñera’s study, survival dropped only slightly in crowded groups (31%–33%) compared to isolated nymphs (41%). In contrast, constant illumination had a much stronger effect, reducing survival from 52% to 21%, despite little change in phenoloxidase levels (Lindberg et al., 2025; Piñera, 2012). These findings indicate that crowding and light stress can modulate immune responses and affect survival through distinct mechanisms. This sensitivity highlights the need to optimise rearing conditions in space, including feed quality and population density. Sex-based immune differences further complicate health dynamics in populations. Males of *A. domesticus* are more susceptible to the bacteria *Serratia liquefaciens* infection than females at equivalent doses (Gray, 1998), implying that sex ratios are relevant in BLSS. Additionally, immune activation is costly: elevated investment may impair female growth and reproduction (Bascuñán-García et al., 2010), suggesting trade-offs between immunity and reproduction. Moreover, immune support may extend beyond physiology. Behavioural immunity, including thermoregulatory “fever” responses observed in *A. domesticus* (Adamo, 1998), and chemical cues used in mate selection may contribute to pathogen avoidance and reproductive success. Chitin, a major exoskeletal component, can stimulate immune activity in insects and potentially also in human consumers and offer an increased gut immunity (Muzzarelli et al., 2012). Lastly, while foundational insights from *D. melanogaster* have informed our understanding of space-altered immunity (Taylor et al., 2014; Iyer et al., 2022), species-specific studies remain essential due to divergent ecological adaptations and immune systems.

## 3 Spaceflight physical stressors: microgravity and radiation

Spaceflight introduces two fundamental physical challenges - microgravity and radiation - that significantly alter insect immunity and pathogen dynamics. These stressors not only compromise the insect host’s immune capacity but may also enhance microbial virulence, creating a dual vulnerability that can jeopardise population stability in Bioregenerative Life Support Systems.

### 3.1 Microgravity-induced immune modulation

Microgravity presents a significant challenge to insect immunity, despite its robustness under terrestrial conditions (Berggren et al., 2025). In *D. melanogaster*, immune function is consistently suppressed following spaceflight. After 13 days in orbit, Iyer et al. (2022) reported broad downregulation of immune genes and impaired cellular responses, reducing both signalling capacity and effector function. Similarly, Marcu et al. (2011) found that although space-reared flies could clear *Escherichia coli* upon return to Earth, immune suppression during flight indicated a temporary vulnerability with potential relevance for BLSS conditions. Exposure to fungal pathogens further revealed a sharp decline in the production of key antifungal peptides, including Drosomycin and Metchnikowin (Taylor et al., 2014). Additional work showed that space-raised larvae had approximately 25% fewer actively phagocytosing plasmatocytes (Marcu et al., 2011), and those cells exhibited altered development and reduced pathogen-engulfing capacity after flight (Kaur et al., 2005). Compounding these immune deficits, altered gravity also increases intracellular reactive-oxygen species (ROS), contributing to oxidative stress (Bhattacharya and Hosamani, 2015). At the same time, elevated levels of heat-shock proteins suggest broader stress-induced modulation of immune regulation and inflammatory control (Shepard et al., 2018). In contrast to microgravity’s suppressive effects, brief exposure to hypergravity, at both 1 g and 4 g, enhanced fungal resistance and improved survival, indicating stress-induced immune priming (Taylor et al., 2014). Together, these findings highlight the critical role of gravitational forces in shaping insect immune function. Circadian disruption may compound immune stress. In *A. domesticus*, constant light nearly halved survival without increasing basal phenoloxidase levels, a pattern also observed under dim night-time lighting in *Teleogryllus commodus* (Piñera, 2012; Durrant et al., 2020). Space-flown *Drosophila* similarly become arrhythmic, downregulate clock genes and exhibit combined metabolic and immune costs (Iyer et al., 2022). Until clinostat data and more knowledge on edible species become available, BLSS colonies may benefit from maintaining a 12 h:12 h light–dark cycle and where feasible to help preserve circadian regulation. Additional immune deficits have been observed under real microgravity. In *Drosophila* spaceflight reduced the fraction of actively phagocytosing plasmatocytes and their phagocytic capacity over time and downregulated pattern-recognition receptors, opsonins, lysozymes and AMP/stress genes indicating coordinated cellular and humoral innate-immune impairments (Marcu et al., 2011).

Although research on edible insects remains limited, studies in *A*. *domesticus* suggest that both nerve cell development and neuroimmune interactions may be sensitive to gravity. Exposure of eggs and early-instar larvae to altered gravity disrupted the physiology of position-sensitive interneurons, indicating that early neural development can be gravity-dependent (Horn et al., 2002; Kirschnick et al., 2007). This is significant because insect immune responses are partially regulated through neuroendocrine signalling, linking neural development to immune competence (Goldsworthy et al., 2003; Urbański et al., 2022). Neurosecretory amines, tachykinin peptides (Urbański et al., 2022), and adipokinetic hormone (Goldsworthy et al., 2003) bind to haemocyte receptors and modulate key immune functions such as phagocytosis, nodulation, and melanisation. Gravity-induced changes in neurogenesis could therefore impair immunity by disrupting this neuro-immune axis, highlighting its integrative and bidirectional role in insect health. Collectively, these findings and the broader synthesis of Berggren et al. (2025), reveal that microgravity disrupts both immune balance and cellular maturation, potentially compromising insect population viability in space. Further research in BLSS-relevant species is urgently needed to determine species-specific thresholds and to guide the development for systems for long-duration missions.

### 3.2 Radiation-induced immune suppression and pathogen synergy

In parallel with microgravity, ionising radiation presents a second major environmental threat to insect health. Space-relevant radiation includes chronic exposure to galactic cosmic rays and solar particle events, both capable of inducing cellular damage and immunosuppression. Evidence from cotton leafworm *Spodoptera littolaris* larvae shows that exposure to gamma radiation at 50, 100, and 150 Gy leads to a dose-dependent decline in key immune enzymes, including phenoloxidase and lysozyme (Gabarty et al., 2013). These effects were significantly exacerbated in the presence of fungal infection, highlighting a synergistic interaction between physical stress and pathogen pressure. Radiation also affects the pathogens themselves. A single high-charge, high-energy particle reduced *Bacillus subtilis* spore germination by more than 90%, while kilogray-level gamma irradiation nearly inactivated *Trichoplusia ni* nucleopolyhedrovirus and greatly reduced the viability of *Beauveria* and *Metarhizium* (Jaques, 1968; Bücker and Horneck, 1975; Rodrigues et al., 2016). These findings suggest that cosmic rays and solar particles could lessen pathogen pressure in space-based insect farms. However, dose–response data remain scarce for most microbiome and pathogen taxa, making it difficult to predict ecological outcomes under long-term exposure. This dual impact, on both host immunity and pathogen viability, may be particularly consequential in BLSS, where containment of insect pathogens is essential.

Overall, radiation weakens insect immunity at both humoral and cellular levels, potentially increasing vulnerability to otherwise non-lethal infections. Species vary in their sensitivity to radiation, underscoring the need for targeted shielding strategies and careful selection of candidate species. While most current data derive from short-term exposures, chronic low-dose radiation in orbit or on planetary surfaces may have cumulative effects on immune function and transgenerational health. Future research should examine how space radiation effects physical and behavioural responses in edible insects as well as microbiome communities. Integrating shielding, dosimetry and biological monitoring into habitat design will be essential for maintaining insect health and ensuring the long-term viability of insect-based food systems.

## 4 Pathogen dynamics in edible insect production: interactions with environmental stressors

Edible insects offer a sustainable protein source for both Earth and space applications, but the rearing under high-density and controlled conditions mean that they have significant disease risks. Insects are vulnerable to a diverse array of pathogens: including viruses, bacteria, fungi, microsporidia, and gregarines, that can lead to population collapse and production loss (Eilenberg et al., 2015; Lecocq et al., 2019; Maciel-Vergara et al., 2021). Although these pathogens do not infect humans directly, their effects on population health and productivity mean a great need for rigorous disease management in both terrestrial and space-based systems.

### 4.1 Viral pathogens and host vulnerability

Viruses represent one of the most severe threats to insect rearing. Outbreaks of *Acheta domesticus* densovirus (AdDV) have caused catastrophic population losses (Maciel-Vergara and Ros, 2017), and recent studies continue to reveal novel viral agents, including iflaviruses in both wild and captive crickets (de Miranda et al., 2021a; de Miranda et al., 2021b). A novel densovirus have also caused high mortality in a commercially reared *T. molitor* (Armién et al., 2023). These viruses persist within dense rearing systems, often amplified through close contact and shared environments. In black soldier flies, Pienaar et al. (2022) documented both current and historic viral interactions, illustrating how rearing conditions shape virome evolution. These findings underscore the need for robust viral surveillance and biosecurity in insect rearing, especially where high density is a norm.

### 4.2 Bacterial threats and environmental entry routes

Bacteria infiltrate insect colonies via contaminated feed or environments and can induce systemic infections. *Pseudomonas aeruginosa*, for example, spreads effectively in dense populations, especially through cannibalism and contact with contaminated frass (Maciel-Vergara et al., 2018). Additionally, insects may carry zoonotic bacteria like *Salmonella spp*. and *Listeria monocytogenes* when raised on infected feed, posing indirect food safety risks (Osimani et al., 2018; van der Fels-Klerx et al., 2018). These outcomes highlight the importance of hygienic feed and controlled environment in the mitigation of pathogens.

### 4.3 Fungal and protozoan infections in confined systems

Entomopathogenic fungi, including *Beauveria bassiana* and *Metarhizium anisopliae*, are frequent in *T. molitor* rearing environments and exploit humid, unsanitised conditions and cause mortality via cuticle penetration (Eilenberg et al., 2015; Lecocq et al., 2019). Similarly, microsporidia and gregarines, though less frequently reported, infect tissues and disrupt reproduction, particularly under stress or poor sanitation (Maciel-Vergara et al., 2021). These parasites remain underdiagnosed due to limited detection tools, yet may become increasingly relevant in compact, resource-limited habitats like those in BLSS.

### 4.4 Density as a central driver of transmission and pathogen evolution

Among all controllable variables in insect farming, population density stands out as a dominant factor influencing disease emergence and spread. Crowded conditions elevate pathogen transmission through increased physical contact, coprophagy, and cannibalism. In *A. domesticus*, high-density rearing has been linked to repeated outbreaks of *Acheta domesticus densovirus* (AdDV), invertebrate iridovirus 6 (IIV-6), and novel iflaviruses, all of which can persist and spread via direct contact and contaminated substrates (de Miranda et al., 2021a; de Miranda et al., 2021b). Virome analyses also show divergence between wild and cultivated cricket virus strains, possibly reflecting evolutionary pressures in commercial systems that favour enhanced transmissibility (de Miranda et al., 2021b). Cannibalism in the giant mealworm *Zophobas morio* has proven an efficient transmission route for *Pseudomonas aeruginosa* and other pathogens under suboptimal hygiene (Maciel-Vergara et al., 2018). These findings reinforce the need for biosecurity protocols tailored to high-density conditions. When individuals are kept close together it may be harder to detect infections, especially when symptoms are mild, like in AdDV-infected crickets that show less movement (Low et al., 2022). While density’s role in pathogen dynamics is clear, its broader implications for insect health and behaviour extend beyond disease ecology.

### 4.5 Nutritional modulation of pathogen susceptibility

Nutritional quality plays a subtle yet critical role in shaping insect susceptibility to infection. Feed composition influences immune function and microbial carriage: nutrient-poor diets in *A*. *domesticus* and *T*. *molitor* reduce protein levels, disrupt fat balance, and impair immunocompetence (Harsányi et al., 2020). In *Hermetia illucens* larvae, diet composition significantly alters gut microbiota, suggesting that tailored feed formulations can support microbial functions linked to digestion and larval health (Li et al., 2024). Protein availability also modulates defence. In *Spodoptera littoralis* challenged with nucleopolyhedrovirus, protein-biased diets improved survival and elevated constitutive immunity (lysozyme-like activity, encapsulation and phenoloxidase), and infected survivors actively selected more protein-rich foods - consistent with protein costs of resistance (Lee et al., 2006). By contrast, in adult *Drosophila* infected with *Micrococcus luteus*, flies reduced protein intake and survival improved on a low-protein, high-carbohydrate diet, with a simultaneous increase in the baseline production of antimicrobial peptides - showing that the best protein-to-carbohydrate ratio for immunity depends on context and life stage (Ponton et al., 2020) Among nutritional components, lipids play a particularly important immunomodulatory role. Balanced lipid profiles enhance immune performance by improving haemocyte membrane fluidity, which promotes receptor clustering and phenoloxidase-mediated pathogen clearance (Li et al., 2023). Specific fatty acids also serve as precursors for eicosanoids, lipid-derived signalling molecules essential for nodulation, haemocyte spreading and antimicrobial peptide production (Kim and Stanley, 2021). These effects are supported by diets with appropriate ratios of polyunsaturated to saturated fatty acids (Orkusz et al., 2024). Finally, feed hygiene also contributes to immune resilience: a recent systematic review found low *Salmonella* prevalence in farmed insects, linking pathogen suppression to clean feed and substrates (Marzoli et al., 2024). Together, these findings underscore the dual role of feed in supporting both growth and disease resistance.

### 4.6 Mitigation strategies: integrated controls for pathogen management

To safeguard insect production systems, especially in BLSS, integrated biosecurity protocols are essential. Core measures include sanitation of facilities, pathogen screening (particularly for viruses and zoonotic bacteria), and quarantining of new stock (Semberg et al., 2018; de Miranda et al., 2021a; de Miranda et al., 2021b). Environmental regulation, humidity, temperature, and especially density control is vital for stress reduction and disease suppression (van Huis, 2021). Furthermore, standardised protocols across feed, hygiene, and rearing environments are necessary to ensure resilience and reproducibility in the systems (Berggren et al., 2019). Emerging biotechnologies may offer additional layers of biosecurity for space-based insect farming. In *Anopheles gambiae*, CRISPR-mediated deletion of the FREP1 gene reduced *Plasmodium* infection by more than 95%, and when paired with a male-biasing gene drive, the trait reached fixation within approximately ten generations (Dong et al., 2018; Simoni et al., 2020). Similar contained genetic modifications could eventually be used to enhance pathogen resistance in edible insect colonies.

## 5 Rearing density and pathogen dynamics

In space-based insect production, rearing density is a central design parameter due to spatial constraints and the drive for high-efficiency biomass generation. However, elevated density introduces significant risks to insect health by amplifying pathogen transmission, altering behaviour, and intensifying stress. These effects are well-documented across terrestrial insect farming and are likely to be exacerbated in confined BLSS, where environmental buffering is limited. Beyond facilitating transmission, high-density rearing environments can shape pathogen evolution. The selection for faster-replicating or more virulent strains in dense populations poses long-term biosecurity concerns, particularly in BLSS where microbial escape and containment options are limited. In *A. domesticus*, producers define overcrowding not by biomass alone, but by physiological stress responses. Laboratory trials with approximately 30 nymphs per 0.5 L enclosure showed elevated phenoloxidase levels, indicating immune strain (Piñera, 2012). On European farms, outbreaks of densovirus and iflavirus have been linked to high-contact rearing conditions, where frass accumulation and cannibalism facilitate viral transmission (de Miranda et al., 2021a; de Miranda et al., 2021b). At comparable densities, *T. molitor* are prone to overheating and fungal epizootics when ventilation is inadequate (Eilenberg et al., 2015; Maciel-Vergara et al., 2021). Microgravity may exacerbate these risks by impairing air exchange. To mitigate potential effects, BLSS insect colonies may benefit from initiating stocking at no more than 80% of terrestrial density, with continuous monitoring of respiration and behaviour to detect early signs of distress (Low et al., 2022). However, the lack of studies in this area makes predictions difficult. Compounding these issues, behavioural changes linked to infection such as reduced activity or delayed emergence observed in AdDV-infected crickets, can reduce behavioural variability within colonies, making it more difficult to detect early signs of illness (Low et al., 2022). This phenomenon presents a diagnostic challenge, especially in systems lacking continuous monitoring. Moreover, density-related stress may suppress behavioural immunity, the natural defence mechanisms like grooming, avoidance, or thermoregulation that help insects mitigate pathogen exposure. Confinement reduces space for these behaviours to manifest, further increasing disease vulnerability. Effective density management, therefore, must go beyond avoiding overcrowding; it must actively support behavioural expression, hygiene, and population health surveillance. To mitigate these risks, a greater understanding of density affects for insect species based on their immune profiles, pathogen susceptibility, and behavioural ecology is needed. Environmental enrichment, optimised feed allocation, and harvesting methods may reduce stress and disease pressure. Integrating density-sensitive biosensors and behaviour-recognition systems may also increase the possibility to detect outbreaks early and enable proactive management.

## 6 Natural behaviours as immunological buffers

While insect immunity is often framed in physiological terms, behavioural and transgenerational adaptations play equally vital roles in sustaining health and resilience, particularly under the multiple stressors of space habitats. In confined and resource-limited systems like BLSS, these behaviours may serve as crucial front-line defences, complementing or even compensating for physiologically weakened immunity due to microgravity, radiation, or nutritional constraints.

### 6.1 Behavioural immunity and environmental navigation

Behavioural immunity encompasses pathogen avoidance behaviours such as grooming, thermoregulation, anorexia, and selective oviposition, all of which reduce pathogen exposure and infection risk (de Roode and Lefèvre, 2012). For example, *A. domesticus* displays fever-like thermoregulatory behaviour in response to intracellular infection, demonstrating a capacity for adaptive thermal regulation in immune defence (Adamo, 1998). Similarly, *T. molitor* larvae can detect and avoid faeces contaminated with pathogen-contaminated feed (Vigneron et al., 2019; Goerlinger et al., 2024). Beyond avoidance, insects can modify mating and foraging behaviours based on immune status or pathogen load. Male beetles may learn to avoid contaminated substrates, while females may exhibit selective mate choice based on chemical cues reflecting immune competence. Infection-driven behavioural shifts such as anorexia, lethargy, or altered movement patterns are not merely symptomatic but adaptive responses that reallocate energy toward immune functions (Adamo, 2006). These behaviours are regulated by hormones and connects the immune system to behaviours (Nunes et al., 2021). However, spaceflight conditions such as reduced gravity and confinement, may suppress these responses, limiting insects’ ability to perform protective behaviours and thereby elevate the infection risk.

### 6.2 Transgenerational immune priming

In addition to real-time behavioural defences, some insects exhibit transgenerational immune priming (TGIP), where parental immune experience enhances the immune function of offspring. In the greater wax moth *Galleria mellonella*, maternal ingestion of bacteria leads to the deposition of bacterial fragments into eggs, triggering upregulation of immune-related genes in the progeny (Freitak et al., 2014). TGIP appears to be underpinned by heritable epigenetic changes. In *Manduca sexta*, parental exposure to bacterial challenge altered DNA methylation and histone acetylation patterns in the offspring, leading to increased expression of immune-related genes (Gegner et al., 2019). Similarly, in bumblebees, primed queens produced daughters with constitutively upregulated antimicrobial and pattern-recognition genes, suggesting an epigenetically “prepared” immune state (Barribeau et al., 2016). Similarly, in *T. molitor*, both maternal and paternal pathogen exposure has been shown to confer elevated pathogen resistance to larvae (Zanchi et al., 2012). TGIP may prove particularly advantageous for multigenerational stability in BLSS, where immune traits acquired in one generation could buffer the next against endemic pathogens or environmental stress. Moreover, the heritability of behavioural immunity and immune investment suggests potential for selective breeding programs to enhance colony robustness over successive generations in space. However, the maintenance of TGIP and behavioural traits under space stressors remain uncertain. Confined conditions, limited mate choice, and altered sensory cues may impair behaviourally mediated disease avoidance. Likewise, chronic exposure to microgravity and radiation may disrupt the stability or effectiveness of inherited immune traits. Understanding how these factors interact with immune memory and behaviour is essential for engineering resilient insect colonies. Maintaining environments that support species-specific behaviours through spatial design, feed and temperature control, may help preserve these natural immune buffers. Further research into genetics and environmental constraints of behavioural and transgenerational immunity will be key to ensuring long-term insect health.

## 7 Integrated system-level responses: monitoring and managing insect health

Maintaining insect health in Bioregenerative Life Support Systems requires continuous, adaptive monitoring capable of detecting physiological stress, disease outbreaks, and environmental imbalances before they escalate. Given the operational constraints of space missions including limited crew time, isolation, and system autonomy; biosensors, AI-driven diagnostics, and automation could be valuable components in resilient insect rearing systems. These tools would serve as an operational interface between insect health and environmental management, helping to detect deviations across all five key stress axes: microgravity, radiation, nutrition, density, and behavioural suppression. Biosensors capable of detecting volatile organic compounds, changes in humidity or temperature, or fluctuations in activity levels could provide early-warning signals of population distress or pathogen presence (Li et al., 2021; Asiri et al., 2025). For instance, disease progression in insects is often accompanied by changes in odour profiles, metabolic rates, or thermal regulation - all parameters detectable by electrochemical or optical sensors. When deployed in enclosed habitats, these non-invasive technologies could enable real-time surveillance. Hand-held qPCR units, flight-tested aboard the International Space Station, can detect *Salmonella enterica* and other pathogens directly from feed, water, or surface swabs in under an hour (Khodadad et al., 2021). Complementary CRISPR/Cas12a-based biosensors have also been developed to identify *Listeria monocytogenes* in insect powders, making them suitable for continuous monitoring of frass and feed (Li et al., 2021). When integrated with optical and AI-based surveillance systems, these lightweight diagnostic tools could form part of a fully automated early-warning system for BLSS insect rearing. Deep learning frameworks are already widely used in insect detection, as shown in a recent review of over 90 studies (Teixeira et al., 2023), and could be adapted to support health monitoring in space-optimised production systems. These platforms could automate alerts for density thresholds, behavioural suppression, or suboptimal environmental conditions that would allow for rapid corrective actions. While promising, the technological readiness of many sensor and AI systems remains variable. Space deployment requires miniaturization, low energy consumption, and autonomous functionality, which are not always compatible with current commercial platforms. Nonetheless, modular diagnostics embedded into rearing infrastructure represent a scalable solution for ensuring the real-time health of insect populations. To fully operationalise these technologies, sensor outputs must be tightly coupled with rearing protocols. For instance, behavioural deviations might trigger automated adjustments in light cycles, substrate distribution, or ventilation, while biosensor alerts could initiate diagnostic imaging or quarantine of affected units. As insect-based BLSS become more complex and multigenerational, system-level monitoring will become increasingly central not just to detect disease, but to ensure long-term viability, optimise productivity and support ecological processes. These technologies will likely form the backbone of precision bioengineering for insect health in space.

## 8 Research gaps and future directions

Despite advances in insect physiology, ecology, microbiology and space systems design, significant knowledge gaps remain across the five key stressors that shape insect health and disease dynamics in BLSS. Addressing these gaps is essential for ensuring the long-term viability of insect-based bioregenerative systems for space exploration.

### 8.1 Microgravity and immune competence

Microgravity-induced immune suppression is one of the most well-documented yet poorly understood challenges. *D. melanogaster* studies reveal downregulated immune gene expression and diminished cellular immunity following spaceflight (Iyer et al., 2022), but equivalent data in edible insect species is lacking. Comparative studies are needed to understand how microgravity alters immune system development, behaviour, and pathogen resistance across insect taxa. Investigating gravity-sensitive phases such as embryogenesis and moulting may yield insights into critical windows for immune disruption. Furthermore, the reversibility of space-induced immunosuppression, as suggested by Marcu et al. (2011), warrants systematic evaluation under BLSS conditions.

### 8.2 Radiation and pathogen interactions

Space radiation presents a cumulative threat to insect immune systems, with few studies addressing long-term, low-dose exposures relevant to orbital or planetary environments. Existing research in *S. littolaris* has shown gamma radiation suppresses phenoloxidase and lysozyme activity, especially when paired with fungal pathogens (Gabarty et al., 2013), but mechanisms remain unclear. Moreover, radiation’s impact on microbiome stability and transgenerational immune function is virtually unexplored. Future studies should simulate galactic cosmic rays and solar particle events to evaluate chronic impacts on insect health and virome evolution in enclosed systems.

### 8.3 Nutrition and microbial ecosystem design

Nutritional inputs affect not only insect growth and productivity but also immune robustness and microbiota composition. However, the link between diet, microbial balance, and immune outcomes in space remains speculative. While nutrient-rich feeds improve lipid profiles and pathogen resistance in *T. molitor* and *A. domesticus* (Harsányi et al., 2020; Orkusz et al., 2024), the effects of sterilised or pre-processed space feeds on gut health and immune development remain unknown. Research should include microbiome-informed feed and probiotic strategies tools to promote gut stability in sterile environments.

### 8.4 Density management and pathogen evolution

High-density rearing accelerates pathogen transmission and may promote virulence evolution. While several studies have shown increased viral loads and behavioural suppression under crowded conditions (de Miranda et al., 2021a; Low et al., 2022), population levels for density-induced stress and infection susceptibility remain undefined. Tools for real-time density diagnostics and automated behavioural monitoring could aid in establishing safe operational ranges. Longitudinal studies of pathogen adaptation under confinement are also needed to assess the risk of virulence escalation and to develop strain monitoring protocols.

### 8.5 Behavioural immunity and heritable resilience

Behavioural adaptations like thermoregulation, grooming, and selective oviposition reduce pathogen exposure, yet these behaviours may be suppressed in confined space habitats. The stability of these behaviours, and of transgenerational immune priming, under microgravity and chronic space stressors is still unknown. While TGIP has been documented in *G. mellonella* and *T. molitor* (Zanchi et al., 2012; Freitak et al., 2014), its persistence across multiple generations in BLSS has not been tested. Research into the genetic foundation for these and how the environmental can affect this is vital for long-term population resilience.

## 9 Conclusion

Insects offer a compelling solution for sustainable protein production and nutrient recycling in space, particularly within Bioregenerative Life Support Systems. However, their viability in closed-loop habitats hinges not only on productivity, but on the ability to maintain robust immune function and population resilience under a suite of spaceflight-specific stressors. This review identifies five key factors - microgravity, radiation, nutrition, rearing density, and behavioural constraints - that interact to shape insect immunity, pathogen dynamics, and microbial balance in space environments (Figure 1). Microgravity and ionising radiation disrupt immune gene expression, cellular maturation, and host-pathogen interactions, posing fundamental challenges to insect resilience. Nutritional inputs modulate immunity and microbiota composition, while high-density rearing conditions accelerate disease transmission and may select for more virulent pathogens. Compounding these risks, behavioural immunity and transgenerational priming that are two natural buffers against infection, may be suppressed in confined or simplified habitats and weaken long-term population stability. Ensuring the success of insect-based BLSS will require a systems-level approach that integrates environmental design, real-time health monitoring and adaptive management. Biosensors, AI diagnostics and automation are likely very useful tools to detect early signs of stress, optimise conditions, and manage pathogen outbreaks across these five axes. Moreover, the development of predictive models that simulate multi-generational effects and multi-factorial stress interactions will be essential for mission planning and habitat design. Ultimately, insect immune health should not be viewed in isolation, but as a central determinant of ecological stability and nutritional reliability in space. Through interdisciplinary research we can develop adaptive insect farming that are resilient not only to the known constraints of space travel, but to the evolving challenges of life beyond Earth.
